# Antidiabetic Properties and Mechanism of Action of *Gynura procumbens* Water Extract in Streptozotocin-Induced Diabetic Rats

**DOI:** 10.3390/molecules15129008

**Published:** 2010-12-08

**Authors:** Zurina Hassan, Mun Fei Yam, Mariam Ahmad, Ahmad Pauzi M. Yusof

**Affiliations:** 1 School of Pharmaceutical Sciences, Universiti Sains Malaysia, 11800 Minden, Penang, Malaysia; Email: mariam@usm.my (M.A.); 2 Department of Human Anatomy, Faculty of Medicine and Health Sciences, Universiti Putra Malaysia, Serdang 43400, Selangor, Malaysia; Email: yammunfei@yahoo.com (M.F.Y.); 3 College of Health Sciences, Masterskill University, Main Campus, Kemacahaya Street, 9^th^ Mile, Cheras 43200, Selangor, Malaysia; Email: apauzi@gmail.com (A.P.M.Y.)

**Keywords:** *G. procumbens*, antidiabetic, medicinal plant

## Abstract

*Gynura procumbens* (Lour.) Merr (family Compositae) is cultivated in Southeast Asia, especially Indonesia, Malaysia and Thailand, for medicinal purposes. This study evaluated the *in vivo* hypoglycemic properties of the water extract of *G. procumbens* following 14 days of treatment and *in vitro* in RIN-5F cells. Glucose absorption from the intestines and its glucose uptake in abdominal skeletal muscle were assessed. The antidiabetic effect of water extract of *G. procumbens* leaves was investigated in streptozotocin-induced diabetic rats. The intraperitoneal glucose tolerance test (IPGTT) was performed in diabetic rats treated with *G. procumbens* water extract for 14 days. In the IPGTT, blood was collected for insulin and blood glucose measurement. After the IPGTT, the pancreases were collected for immunohistochemical study of β-cells of the islets of Langerhans. The possible antidiabetic mechanisms of *G. procumbens* were assessed through *in vitro* RIN-5F cell study, intestinal glucose absorption and glucose uptake by muscle. The results showed that *G. procumbens* significantly decreased blood glucose levels after 14 days of treatment and improved outcome of the IPGTT. However, *G. procumbens* did not show a significant effect on insulin level either in the *in vivo* test or the *in vitro* RIN-5F cell culture study. *G. procumbens* also showed minimal effects on β-cells of the islets of Langerhans in the pancreas. However, *G. procumbens* only significantly increased glucose uptake by muscle tissues. From the findings we can conclude that *G. procumbens* water extract exerted its hypoglycemic effect by promoting glucose uptake by muscles.

## 1. Introduction

There is a variety of glucose-lowering agents available for the treatment of type 2 diabetes with differing mechanisms of action, although side effects, including weight gain and the risk of hypoglycemia, have been the main obstacles hindering achievement of glycemic targets. This treatment gap is highlighted by the recent controversy surrounding the outcome of the Action to Control Cardiovascular Risk in Diabetes (ACCORD) study, in which subjects who received intensive glucose control had increased weight gain, increased risk of hypoglycemia and increased risk of mortality during the study [1]. Thus, new strategies are needed for the prevention and treatment of diabetes. Among the best existing alternative therapies are herbal remedies, which have been used since ancient times for the treatment of diabetes mellitus [2]. About 90% of the population in rural areas of developing countries relies solely on traditional medicines for their primary health care. 

*Gynura procumbens* (Lour.) Merr (family Compositae), also known locally as “Sambung Nyawa”, is cultivated in Southeast Asia, especially Indonesia, Malaysia and Thailand, for medicinal purposes. This plant is reported to be useful for hypertension, anti-inflammation, anti-herpes simplex virus, prevention of rheumatism, and treatment of eruptive fevers, kidney troubles, colon cancer, hemorrhoids and diabetes [3]. However, little information about the mechanism of action involved in the antidiabetic activity of *G. procumbens* is available. The aim of the present studies was to evaluate the hypoglycemic properties of the water extract of *G. procumbens in vivo* and to investigate its possible antidiabetic mechanisms.

## 2. Results and Discussion

### 2.1. Results

#### 2.1.1. Effects of *G. procumbens* water extract on body weight of streptozotocin-induced diabetic rats after 14-day treatment

Table 1 shows the effects of *G. procumbens* water extract (500 and 1,000 mg/kg), metformin and (normal saline) control treatment on body weight changes in streptozotocin-induced diabetic rats. After 14-day treatment with the water extracts, metformin and normal saline, body weight was reduced significantly from day 0 (before treatment) to day 14 (after treatment) (*P* < 0.05).

**Table 1:** Effects of *G. procumbens* water extract and metformin on body weight before and after 14 days treatment in diabetic rats.

Experimental groups	Dose (mg/kg)	Body weight (g)	
		Day 0	Day 14
Control		240.4 ± 16.5	202.6 ± 16.4^*^
Metformin	500	223.5 ± 13.1	189.0 ± 9.4^***^
*G. procumbens* water extract	500	204.4 ± 12.8	162.4 ± 11.5^***^
*G. procumbens* water extract	1000	206.0 ± 5.4	167.4 ± 5.2^***^

Each value represents the mean ± S.E.M (n = 5); * and *** indicate significant differences between day 0 and day 14 of same treatment group at *P* < 0.05 and *P* < 0.001, respectively.

#### 2.1.2. Effects of *G. procumbens* water extract on fasting blood glucose levels in streptozotocin-induced diabetic rats after 14-day treatment

**Figure 1:**
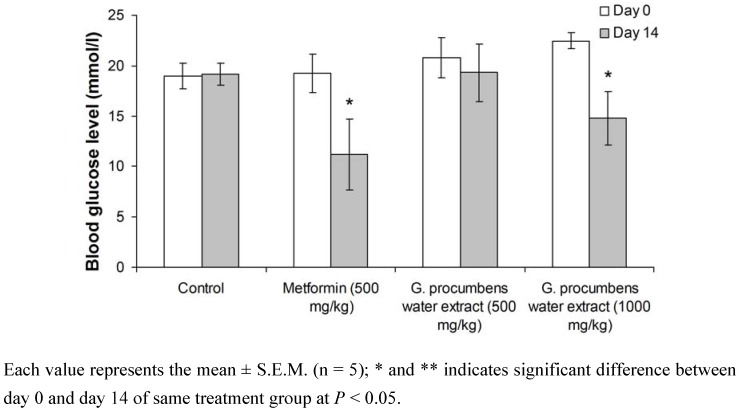
Comparison of fasting blood glucose levels of water extract of *G. procumbens* and metformin before and after 14-day treatment in diabetic rats.

The hypoglycemic effect of repeated oral administration of the *G. procumbens* water extract in diabetic rats is shown in Figure 1. After two weeks, streptozotocin-induced diabetic rats that received *G. procumbens* water extract (1,000 mg/kg) and metformin had significantly decreased fasting blood glucose levels at *P* < 0.05.

#### 2.1.3. Effects of *G. procumbens* water extract on the plasma insulin levels in diabetic rats after 14 days of treatment

As shown in Table 2, the plasma insulin levels did not differ after repeated oral treatment with metformin or water extract (500 and 1,000 mg/kg) in streptozotocin-induced diabetic rats. No significant changes in the plasma insulin levels were found between the control group and treated-diabetic rats, either before or after treatment.

**Table 2:** Effect of *G. procumbens* water extract and metformin on plasma insulin level in streptozotocin-induced diabetic rats.

Experimental groups	Dose (mg/kg)	Insulin concentration (ng/mL)	
		Day 0	Day 14
Control		2.49 ± 0.11	2.31 ± 0.03
Metformin	500	2.17 ± 0.06	2.11 ± 0.05
*G. procumbens* water extract	500	2.26 ± 0.06	2.09 ± 0.06^*^
*G. procumbens* water extract	1000	2.21 ± 0.06	2.15 ± 0.03

Each value represents the mean ± S.E.M. (n = 6); * indicate significant differences between day 0 and day 14 of same treatment group at *P* < 0.05.

#### 2.1.4. Effects of *G. procumbens* water extract on IPGTT in streptozotocin-induced diabetic rats after 14 days of treatment

**Figure 2:**
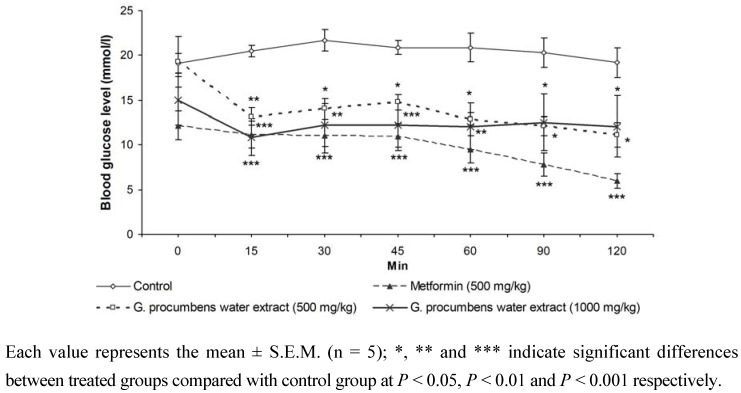
Effect of repeated oral administration of water extracts of *G. procumbens* and metformin on intraperitoneal glucose tolerance test (IPGTT) after 14-day treatment in diabetic rats. Glucose was given intraperitoneally 60 min after the administration of the extract.

Figure 2 shows the effect of repeated oral administration of *G. procumbens* water extract, metformin and normal saline (control) on IPGTT after 14 days of treatment in diabetic rats. For the *G. procumbens* water extract (500 or 1,000 mg/kg)-treated group, the glucose tolerance was significantly improved on day 14 compared with the control group. Similar results were obtained in metformin-treated group; after 14 days of metformin treatment, the glucose tolerance was significantly improved compared with the control group. These data showed that the extract (500 or 1,000 mg/kg)- and metformin-treated groups had significantly increased glucose disposal from 15 to 120 min after glucose load.

#### 2.1.5. Effects of *G. procumbens* water extract on glucose absorption from the intestine

**Figure 3:**
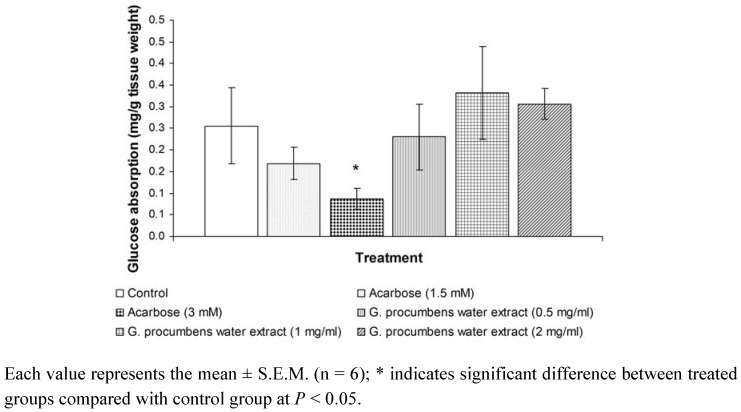
Effects of water extract of *G. procumbens* and acarbose on glucose absorption by everted sac technique.

Figure 3 demonstrates the effect of acarbose and *G. procumbens* water extract on intestinal absorption of glucose in the everted sac segments. Only acarbose (3 mM) produced a significant reduction in intestinal absorption of glucose. This finding confirms that the result can be implemented by demonstrating the inhibitory effect of acarbose on glucose absorption compared with the control group. Treatment with the water extract (0.5, 1 or 2 mg/mL) had no effect on glucose absorption when compared with the control group. Although the effect of *G. procumbens* water extract (1 mg/mL) on glucose absorption (mg/g tissue weight) was quite high, there was no significant difference.

#### 2.1.6. Effects of *G. procumbens* water extract on glucose uptake by isolated rat abdominal muscle

**Figure 4:**
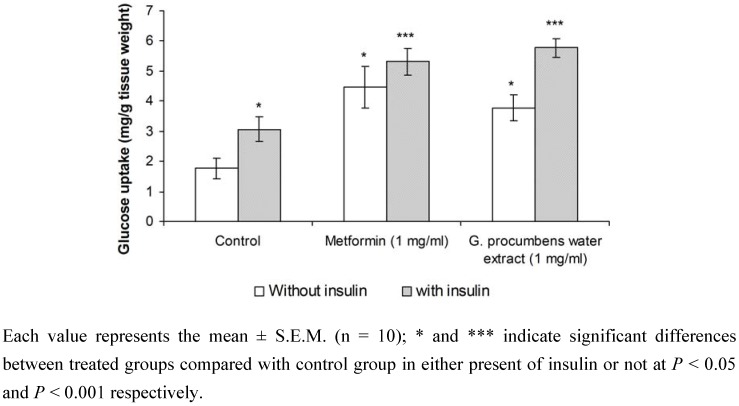
Effect of water extract of *G. procumbens* and metformin on glucose uptake in isolated rat abdominal muscle (in the present or absence of insulin).

Figure 4 shows glucose uptake (mg per g tissue weight) by isolated rat abdominal muscle in the absence and presence of 100 mU/mL insulin. In normal isolated rat abdominal muscle, the absence of insulin in the (Kreb's-Ringer bicarbonate buffer) KRB resulted in a significant increase (*P* < 0.05) in glucose uptake in the metformin (1 mg/mL)-treated group (4.47 ± 0.69 mg per g tissue weight) when compared with the control group (1.76 ± 0.35 mg per g tissue weight). Treatment with *G. procumbens* water extract (1 mg/mL) also elicited a significant increase in glucose uptake by isolated rat abdominal muscle (*P* < 0.05) (3.77 ± 0.43 mg per g tissue weight) when compared with the control in the absence of insulin.

Glucose uptake by isolated rat abdominal muscle from control groups in the presence of insulin (100 mU/mL) was significantly higher (*P* < 0.05) than in the absence of insulin (3.06 ± 0.41 mg per g tissue weight and 1.76 ± 0.35 mg per g tissue weight, respectively). The presence of insulin in the KRB significantly increased the glucose uptake by isolated rat abdominal muscle in metformin- and *G. procumbens* water extract-treated groups (5.32 ± 0.44 mg per g tissue weight and 5.77 ± 0.32 mg per g tissue weight, respectively) (*P* < 0.05) when compared with control and insulin treatment alone.

#### 2.1.7. Effects of *G. procumbens* water extract on regeneration of β-cells in streptozotocin-induced rats

**Figure 5:**
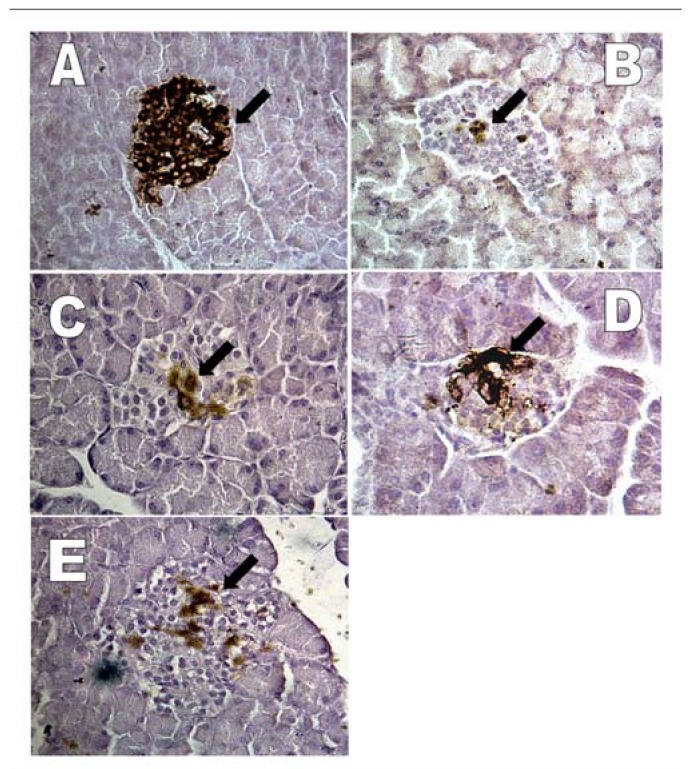
Light micrograph of rat pancreas showing insulin immuno-staining of β-cells (arrow) in islet of Langerhans from A) normal rat, B) 72 h after streptozotocin injection, C) streptozotocin injection and treated with saline, D) streptozotocin injection and treated with metformin, E) streptozotocin injection and treated with water extract of *G. procumbens* (× 400).

Figure 5(A) shows islets with a relatively large area (82% of islets size) of positive immunoreactivity, indicating the presence of insulin in the pancreas of normal rats. As shown in Figure 5(B), there was a small number of insulin-positive cells (2.9% of islets size) 72 h after injection with streptozotocin and before treatment. After 14 days of treatment with normal saline to the streptozotocin-induced diabetic rats, the pancreas showed a relatively small area (8% of islets size) with insulin-positive cells [Figure 5(C)]. In contrast, treatment of streptozotocin-induced diabetic rats with metformin resulted in a large area (21% of islets size) of positive immunoreactivity for the presence of insulin with many brown insulin granules distributed in the β-cells in the islets of Langerhans [Figure 5(D)]. Figure 5(E) shows the area (7% of islets size) with positive immunoreactivity indicating the presence of insulin in the pancreas from diabetic rats treated with *G. procumbens* water extract.

Table 3 shows the percentage of insulin-positive cells in the pancreas of normal rats, 72 h after streptozotocin injection and diabetic rats treated with saline, metformin and *G. procumbens* water extract. The results reveal that insulin-positive cells were found in all groups, with normal rats having the most abundant insulin-positive cells. The difference in the percentage of insulin-positive cells between normal and 72 h after streptozotocin injection animals was highly significant (82.5% and 2.9% respectively, *P* < 0.001). In contrast, when streptozotocin-induced diabetic rats were treated with saline, metformin or *G. procumbens* water extract for 14 days, the percentage of insulin-positive cells increased significantly to 8.4%, 20.9% and 7.0%, respectively, when compared with 72 h after streptozotocin injection (2.9%). These results indicate that *G. procumbens* water extract did not improve the viability of β-cells because there was no significant difference between diabetic rats treated with *G. procumbens* water extract and those treated with saline. Only metformin treatment showed improvement in the viability of β-cells.

**Table 3:** Effect of *G. procumbens* water extract and metformin on islet cell in streptozotocin-induced diabetic rats.

Experimental group	Day of treatment	Percentage of insulin-positive cells per islet
Normal	--	82.52 ± 2.38^###^
72 hours after STZ injection	--	2.93 ± 0.51^***^
Normal saline+STZ injection	14	8.43 ± 0.61^***###^
Metformin (500 mg/kg)+STZ injection	14	20.93 ± 3.46^***###^
*G. procumbens* water extract (1000 mg/kg)+STZ injection	14	7.02 ± 1.14^***##^

Each value represents the mean ± S.E.M (n = 10); ^***^ indicates significant difference as compared to the normal group at *P* < 0.001; ^## ^and ^### ^indicate significant differences as compared to the 72 h after streptozotocin injection group at *P* < 0.01 and *P* < 0.001 respectively. STZ = Streptozotocin.

#### 2.1.8. Effects of *G. procumbens* water extract on insulin secretion by RIN-5F cells

**Figure 6:**
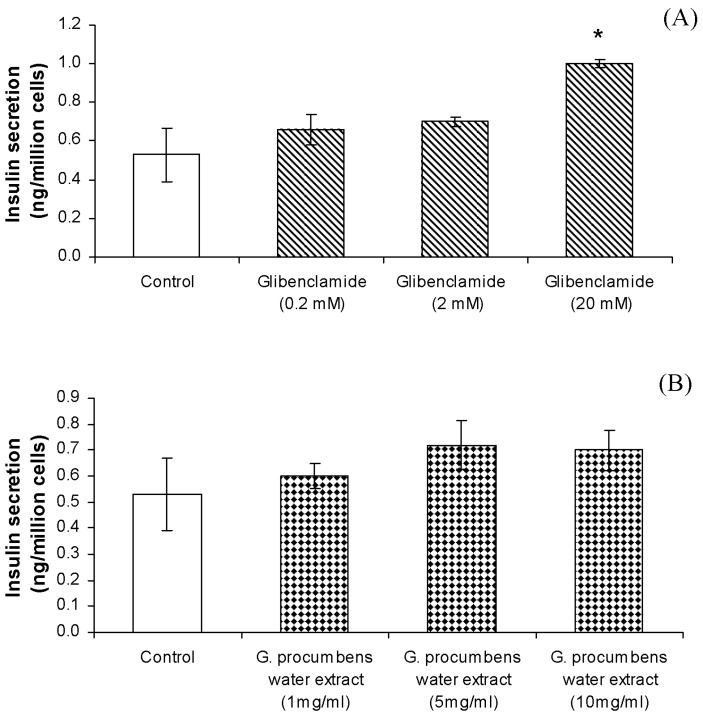
**(A)** Effects of glibenclamide (mM) on insulin secretion by RIN-5F cells. Each value represents the mean ± S.E.M. (n = 6); * indicates significant difference between treated groups compared with control group without glibenclamide at *P* < 0.05. **(B)** Effects of *G. procumbens* water extract on insulin secretion by RIN-5F cells. Each value represents the mean ± S.E.M. (n = 6).

Glibenclamide (0.2-20 mM) produced a dose-dependent stimulatory effect on insulin secretion by RIN-5F cells incubated in 1.1 mM glucose [Figure 6(A)]. RIN-5F cells exposed to 20 mM of glibenclamide for 20 min showed maximal levels of stimulation. However, concentrations of glibenclamide less than 20 mM did not significantly enhance the insulin–releasing effect. As seen in Figure 6(B), treatment of RIN-5F cells with different concentrations of water extract of *G. procumbens* (1-10 mg/mL) did not significantly increase the levels of insulin as compared with the control.

#### 2.1.9. Effects of *G. procumbens* water extract on RIN-5F cell viability

*G. procumbens* water extract at concentrations of 1, 5 and 10 mg/mL showed no cytotoxic effect in RIN-5F cells.

### 2.2. Discussion

This study demonstrated that water extract of *G. procumbens* leaves possesses hypoglycemic activity. As seen in Figure 1, repeated oral administration of the water extract (1,000 mg/kg) or metformin (500 mg/kg) significantly reduced the fasting blood glucose levels after 14 days of treatment, with decreases of 29% and 49%, respectively. However, there was no significant effect of either water extract or metformin on plasma insulin levels (Table 2).

The ability of the extract to improve blood glucose regulation was further investigated by IPGTT. After 14 days of treatment with *G. procumbens* water extract (500 or 1,000 mg/kg) the hyperglycemic peak was significantly reduced (Figure 2). *G. procumbens* water extract (500 and 1,000 mg/kg)-treated rats were able to excess glucose from the blood at a significantly faster rate than the diabetic control animals. Moreover, both metformin and *G. procumbens* water extract (500, 1,000 mg/kg) produced significant clearing of the postprandial glucose as early as 15 min after glucose loading and this effect lasted for 2 h (Figure 2).

To investigate the mechanism of action of *G. procumbens* water extract as a hypoglycemic agent, *in vitro* experiments were conducted. Investigations were conducted on the intestinal level by delaying or inhibiting glucose absorption, the peripheral level on insulin-sensitive tissues by facilitating the entry of glucose into cells such as muscle, and the pancreatic level by stimulating insulin secretion.

Severe postprandial hyperglycemia commonly experienced by diabetics could be prevented if the rate of glucose uptake from the intestine into the circulation could be reduced by inhibiting carbohydrate digestion and absorption. This action results in diminished and delayed rise in postprandial glucose concentration, leading to normal glucose homeostasis in diabetic subjects. Hypoglycemic effects can be due to decreased intestinal glucose absorption provoked by high dietary fiber content [4,5]. Alpha glucosidase is one of the enteric digestive enzymes that degrades polysaccharides into monosaccharides (the only form of glucose that can be absorbed in the intestinal lumen and transported in blood circulation). Acarbose, a clinically available alpha-glucosidase inhibitor, has shown its inhibitory action by reducing the digestion of oligosaccharides in the proximal half of the small intestine by prolonging the absorption of monosaccharides after a meal. Thus, in patients with diabetes, acarbose decreases postprandial hyperglycemia [6]. An acarbose concentration of 1 mg/mL produced the greatest reduction in the intestinal absorption of glucose [6,7]. Furthermore, acarbose has been shown to significantly inhibit maltose absorption at concentrations of 0.2 and 2 mM [8]. In the present study, acarbose significantly reduced glucose absorption at the concentration of 3 mM. However, none of the *G. procumbens* water extracts produced reductions in glucose absorption.

In this study, *in vivo* results showed that *G. procumbens* water extract did not stimulate insulin secretion and inhibited endogenous insulin production. It may be hypothesized that the substance contained in the *G. procumbens* water extract acts at the peripheral level (an insulin-like effect). *G. procumbens* water extract mimicked or enhanced the action of insulin, but the nature and magnitude of the effect depends on the pathway being studied. It has been established that insulin promotes glucose uptake into peripheral cells, and metformin is the only drug currently used for improving insulin action. To further investigate this possibility, *in vitro* experiments were performed by using rat abdominal muscle. Skeletal muscle represents 30-40% of the total body weight and seems to be one of the most important target tissues for the action of insulin and for the uptake of glucose at the peripheral level [9]. Uptake of glucose by the rat abdominal muscle (mg/g tissue weight) was calculated as the clearing of glucose from the perfusion medium. In this study, the *G. procumbens* water extract was found to enhance insulin-stimulated glucose transport across the membrane of skeletal muscle, similar to metformin in the absence of insulin. The glucose uptake was higher in *G. procumbens* water extract with insulin compared to without insulin (Figure 4). Hence, the *G. procumbens* water extract acts directly in the uptake of glucose at the peripheral levels, either alone or as an insulin coadjuvant. Insulin is needed to promote the storage of fat as well as glucose (both sources of energy) within specialized target cells and influence cell growth and the metabolic functions of a wide variety of tissues.

Streptozotocin has been shown to cause direct irreversible damage to β-cells of pancreatic islets of Langerhans, resulting in degranulation and loss of insulin secretion. Clarification of the regenerating potential in experimentally-induced diabetic animals would be of interest as an alternative therapy for diabetes [10]. The distribution and number of functioning β-cells in the islets using immunohistochemical methods were studied. The results of this study demonstrated marked changes in the distribution pattern of insulin-positive cells in pancreatic tissue of diabetic rats compared with that of normal rats (Figure 5). The number of insulin-positive cells decreased markedly in both treated and untreated diabetic rats compared with normal rats (Table 3). However, the percentage of insulin-positive cells per islet was significantly increased (*P <* 0.001) in the metformin-treated diabetic rats when compared with saline-treated diabetic rats. In comparison, there was no significant difference in the number of insulin-positive cells in the water extract-treated diabetic rats when compared with saline-treated diabetic rats. This observation indicates that metformin may play an important role in increasing the number of insulin-positive cells in the pancreas. There are two possible explanations for these findings. First, metformin may exert its effect by preventing the destruction of β-cells of the islets of Langerhans. Second, metformin may trigger the recovery of partially destroyed β-cells. It is possible that metformin initiates cell proliferation because it has been reported that pancreatic endocrine cells have the potential to proliferate after induction of diabetes with streptozotocin [10]. In contrast, the *G. procumbens* water extract does not improve the viability of β-cells. The results showed no significant increase in plasma insulin levels in streptozotocin-induced diabetic rats after 14 days of oral treatment with *G. procumbens* water extract (Table 2). In addition, *G. procumbens* water extract treatment did not activate β-cells of the pancreas as demonstrated by the minimal immunohistochemical staining for insulin of the pancreas of *G. procumbens* water extract treated rats (7% of islets cells were positive) (Table 3). Although insulin immunoreactivity was observed in the pancreatic tissues of metformin treated rats (20.9% of islets cells were positive), the plasma insulin levels in streptozotocin-induced diabetic rats after 14 days of oral treatment with metformin was not significantly different from streptozotocin-induced diabetic rats treated with saline. This finding may be explained by the fact that metformin does not depend on a functional pancreas for its activity.

The *in vivo* results indicate that the mechanism by which this plant decreases blood glucose levels in diabetic rats is independent of insulin secretion. This was further studied by examining the insulin-secreting property of the extract *in vitro*. Exposure of cloned pancreatic β-cells, the RIN-5F cell line, to various concentrations of water extract showed no stimulation of insulin secretion. These results support the *in vivo* experiments, providing evidence that the hypoglycemic action of the extract does not rely on insulin secretion. Glibenclamide, a sulphonylurea derivative, was shown to evoke a dose-dependent stimulation of basal insulin release from the RIN-5F cell line. At the dose of 20 mM, glibenclamide was found to stimulate insulin secretion. 

In conclusion, the antihyperglycemic effect of *G. procumbens* water extract may be due to its ability to mimic or improve insulin action at the cellular level. This could be an insulin-like effect of the active component in the extract. A preliminary phytochemical analysis of the *G. procumbens* water extract led to isolation of flavonol and flavonol glycosides, including rutin, quercetin and kaempferol [11]. In addition, high performance thin layer chromatography (HPTLC) qualitative analysis revealed that the water extract of *G. procumbens* contains 0.76% and 2.65% of kaempferol-3-O-rutinoside and astragalin, respectively. These flavonoids and their glycosides have been found to be responsible for blood glucose lowering activity [12].

## 3. Experimental

### 3.1. Preparation of G. procumbens extract

Leaves of *G. procumbens* (L.) Merr. (Compositae) were collected at the Malaysian Research on Agriculture Development Institute (MARDI), Kepala Batas, Penang, Malaysia, and identified. A voucher specimen (10117) was deposited in the herbarium of the School of Biological Sciences. The leaves were dried in an oven at 40 °C and milled into powder. The dry powdered leaves, weighing 162.5 g, were extracted with distilled water (3 L) for 24 h under reflux and heated to 60 °C. The extract was filtered with cotton wool and concentrated at 55 °C using a rotary evaporator (Buchi Labortechnik AG, Switzerland). The extract was then freeze-dried to yield a water extract of 47.5 g, which was then dissolved in normal saline before use. The amount of kaempferol-3-O-rutinoside and astragalin in the *G. procumbens* water extract were determined using Camag densitometry (Camag Model-3 TLC scanner equipped with Camag CATS 4 software), as previously described [13]. The *G. procumbens* water extract contained 0.76% and 2.65% of kaempferol-3-O-rutinoside and astragalin, respectively.

### 3.2. Animals

Male Sprague-Dawley (SD) rats (200-250 g) were obtained from the animal house of the School of Pharmaceutical Sciences, Universiti Sains Malaysia, Penang. They were housed in standard environmental conditions (24 ± 1 °C) with 12 h light: 12 h dark cycles and fed a commercial diet and water *ad libitum.*

### 3.3. Streptozotocin-induced diabetic rats

Diabetes was induced by intraperitoneal injection of streptozotocin (Sigma, USA) (65 mg/kg body weight in 0.9% NaCl, pH 4.5) to rats fasted for 16 h. Their diabetic conditions were confirmed by the symptoms of polydipsia, polyuria and a high fasting blood glucose concentration 72 h after injection of streptozotocin. Rats with a blood glucose level above 15.0 mmol/L were considered to be diabetic and used in the experiment.

### 3.4. 14 day treatment with the extracts

Diabetic rats (n = 5) were treated orally with the water extract (500 or 1,000 mg/kg) twice daily (at 9.00 am and 9.00 pm) for 14 days. Another group (n = 5) was given oral saline (10 mL/kg) and served as controls. Metformin (500 mg/kg) was given as a positive control. The body weight of the rats was recorded daily. At the end of the experimental period, blood samples were collected for the measurement of blood glucose level and plasma insulin. An intraperitoneal glucose tolerance test (IPGTT) was performed at the end of 14-day treatment [14].

### 3.5. Intraperitoneal glucose tolerance test (IPGTT)

The IPGTT was conducted at the end of 14-day treatment after rats had been fasted overnight (at least 16 h). The rats were divided into four groups, each group consisting of five animals, as follows:

Group I     Diabetic rats given oral saline (10 mL/kg)
Group II    Diabetic rats given *G. procumbens* water extract (500 mg/kg)
Group III   Diabetic rats given *G. procumbens* water extract (1,000 mg/kg)
Group IV   Diabetic rats given metformin (500 mg/kg)

Glucose (500 mg/kg) was administered intraperitoneally 60 min after treatment. Blood samples were collected from the tail vein at time –60 min (just before the administration of the extract by gastric intubation), time 0 (prior to the glucose load) and 15, 30, 45, 60, 90 and 120 min after the glucose load. Blood glucose concentration was measured using the Accu-check Advantage II Clinical Glucose meter (Roche diagnostics Co. USA) [14].

### 3.6. Preparation of plasma insulin

Blood samples (~0.5 mL) collected from the tail vein using hematocrit-capillary tubes containing Na-heparin (15 units/mL of blood sample) were centrifuged at 12,000 rpm at 4 °C for 3 min. Plasma samples were then stored at –20 °C. Insulin concentrations in the plasma samples were assayed by enzyme-linked immunosorbant assay (ELISA) using a kit purchased from Crystal Chem, USA [15,16].

### 3.7. Measurement of glucose absorption from the intestine

SD rats weighing 200-250 g were sacrificed, and their abdominal walls were dissected. The jejunum (20 cm away from the pylorus) was removed. The isolated jejunum was everted as previously described [17]. The everted part of jejunum was cut into 5-cm long segments and put into oxygenated tyrode solution (342 mM NaCl, 6.7 mM KCl, 5.9 mM CaCl_2_·2H_2_O, 5.3 mM MgCl_2_, 59.5 mM NaHCO_3_, 2.08 mM NaH_2_PO_2_ and 5.5 mM glucose). Each segment was filled with 0.5 mL of tyrode solution and tied at both ends to form a sac. The sac was then incubated in 15 mL of tyrode solution in the presence of test substances and gassed with 95% O_2_ and 5% CO_2_ at 37 °C for 60 min. The test substances were 0.5, 1 and 2 mg/mL of *G. procumbens* water extracts. Acarbose (1.5 and 3 mM), an α-glucosidase inhibitor, was used as a positive control, while tyrode solution served as negative control. The glucose concentration was analyzed using a glucose analyzer (YSI model 23A Sidekick^®^). The concentrations of glucose were measured before and 60 min after incubation, and measurement were made outside of the sacs so that the amount of glucose transported could be calculated. The following calculation was used to calculate the amount of glucose transported into the intestine:

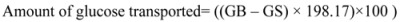

weight of intestine (g); GB = glucose concentration before incubation; GS = glucose concentration outside the sac.

### 3.8. Measurement of glucose uptake by isolated rat abdominal muscle

Glucose uptake by isolated rat abdominal muscle was measured according to Gray and Flatt (1998) with minor modifications [18]. SD rats (200-250 g) were sacrificed, and the abdominal muscle was exposed [18]. The abdominal muscle of both the left and right side were cut into small squares (90-150 mg per muscle) and left in Kreb's-Ringer bicarbonate buffer (KRB; 118 mM NaCl, 5 mM KCl, 1.28 mM CaCl_2_, 1.2 mM KH_2_PO_4_, 1.2 mM MgSO_4_, 25 mM NaHCO_3_) in the presence of 95% O_2_ and 5% CO_2 _at 37 °C. The abdominal muscle was incubated and aerated in KRB solution for 10 min. The KRB solution was then replaced with KRB containing 11.1 mM of glucose, and samples were collected from the KRB solution as a baseline reading. The KRB solution was treated with 1 mg/mL of extract and 1 mg/mL of metformin in the presence or absence of 100 mU/mL insulin (Actrapid, Novo, Copenhagen, Denmark). The KRB solution was aerated for 5 min before incubation for 30 min on a shaker at 96 rpm. Samples were then collected after 30 min, and the muscle weight was measured. Samples used either for baseline measures or after 30 min of treatment were collected and analyzed using a glucose analyzer (YSI model 23A Sidekick^®^) [18,19].

### 3.9. Studies on regeneration of β-cells in streptozotocin-induced rats

#### 3.9.1. Preparation of the parafinized tissues

At the end of the 14th day of treatment, the rats were sacrificed, and the pancreas was removed and fixed in 10% buffered formalin for two days. The pancreatic tissue was then dehydrated with a series of alcohols, xylene and liquid paraffin at 58 °C before being embedded in paraffin. First, the tissues were dehydrated by incubating in 70%, 80%, 90% and 100% ethanol and xylene for 30 min each. Tissues were then incubated in liquid paraffin (58 °C) twice for 60 min each. Finally, the tissues were embedded with an embedding ring.

#### 3.9.2. Immunohistochemical staining for insulin

Paraffin sections were serially cut at 5 μm in thickness and placed on microscope slides that had been sealed with poly-L-Lysine (Sigma, USA). The tissue was fixed on the microscope slide in a 45 °C oven for 1 h. Then, the tissues were deparaffinized by rinsing in xylene three times and dehydrated with two rinses in absolute alcohol and two rinses in 95% ethanol for 3 min each before being washed with phosphate buffer saline (PBS) and distilled water for 5 min each. The tissues sections were then incubated for 15 min in 3% H_2_O_2_ in methanol. This step was done to quench the endogenous peroxides. The sections were then washed in PBS for 5 min and excess PBS was wiped from around the tissues. The sections were blocked by incubating with diluted normal serum for 20 min and excess serum was blotted from the sections. For detection of insulin, sections were incubated with primary antibody (Guinea-pig polyclonal anti-insulin body from Zymed laboratories, San Francisco, CA, USA) diluted to 1:100 in PBS for 30 min, and sections were washed with PBS for 5 min. Excess PBS was wiped from the slides. Then, the sections were incubated with diluted biotinylated secondary antibody solution for 30 min. The slides were then washed with PBS for 5 min. Vectastain ABC (Vector Laboratories, USA) reagent was incubated with the tissues for 30 min. The slides were then washed again for 5 min and excess buffer removed from slides. Sections were then incubated in 3,3-diamino- benzidine tetrahydrochloride (DAB) (DAKO, Japan) for 3 to 5 min at room temperature. Slides were washed three times in distilled water. Then, two to four drops of DAB enhancer was added to cover the tissues on the slides and incubated for 1 to 3 min at room temperature. Color development was monitored under microscope. The sections were rinsed with two to three changes of distilled water. The nuclei were counterstained with Harris Hematoxylin. The tissues were then dehydrated in a graded series of alcohol. The dehydration series consisted of three rinsed in 95% ethanol, two rinses in absolute alcohol and three clearings in xylene for 3 min each. Finally, the tissues were mounted with DPX (BDH, UK). Evaluation of immunohistochemical staining was made by examination of 10 islets for each group of rats using a Leica MZ6 optical microscope (Leica Microskopie und Systeme, Germany) equipped with a Leica Qwin (Leica Imaging Systems, Cambridge, England).

### 3.10. In vitro studies

RIN-5F cells were routinely cultured in RPMI 1640 supplemented with 2 mM L-glutamine, 10 mM HEPES, 1 mM sodium pyruvate, 4.5 g/L glucose, 1.5 g/L sodium bicarbonate and 10% fetal bovine serum. The cells were passaged 2-4 days before each experiment and plated in 24-well Nunclon multiwell plates (NUNC A/S, Denmark) at a density of 0.2 × 10^6^ cells/well. Insulin secretion was measured as previously described by Gray and Flatt (1998, 1999) [18,20]. Multiwells were seeded with 0.2 × 10^6 ^cells and insulin release measured after 4-5 days as follows. Cells were washed three times with KRB (115 mM NaCl, 4.7 mM KCl, 1.28 mM CaCl_2_, 1.2 mM KH_2_PO_4_, 1.2 mM MgSO_4_, 24 mM NaHCO_3_, 10 mM HEPES-free acid, 1 g/L bovine serum albumin, 1.1 mM glucose; pH 7.4) and preincubated for 40 min at 37 °C. Cells were then incubated for 20 min with 1 mL KRB and 1.1 mM glucose in the absence or presence of *G. procumbens* water extract (1, 5 or 10 mg/mL) and glibenclamide (0.2, 2 or 20 mM). Following incubation, aliquots were removed from each well and stored at -20 °C for insulin assay. Insulin release was measured by rat an insulin ELISA kit (Crystal Chem, USA). 

### 3.11. Cytotoxicity assay

The viability of RIN-5F cells after treatment with *G. procumbens* water extract was assayed by the reduction of 3-(4,5-dimethylthiazol-2-yl)-2,5-diphenyltetrazoliumbromide (MTT) to formazan, as previously described [21]. Briefly, cells were seeded in 96-well microtiter plates (1 × 10^4 ^cells/well in 200 μl of medium) and left to adhere to the walls overnight before being exposed to the *G. procumbens* water extract (1, 5 or 10 mg/mL). The plate was then incubated at 37 °C for 24 h. After 24 h exposure to *G. procumbens* water extract, 50 μL of (5 mg/mL) MTT solution was added to each well, and the cells were incubated in the dark at 37 °C for an additional 4 h. Thereafter, the medium was removed, the formazan crystals were dissolved in 200 μL of DMSO and the absorbance was measured at 570 nm in a microplate reader (Power Wave X340, USA).

### 3.12. Statistical analyses

Results were expressed as mean ± standard error of mean (S.E.M.). The blood glucose levels of extract-treated animals, the reference drug-treated and vehicle-treated controls were compared by paired T-tests (compare of glucose and insulin level, and body weight day 0 before treatment and day 14 after treatment), Student’s T-test (compare 2 groups) or one way ANOVAs. Differences with *P* < 0.05 were considered to be statistically significant.

## 4. Conclusions

In conclusion, our *in vivo* studies indicate that the water extract of *G. procumbens* leaves possesses a significant antihyperglycaemic effect in streptozotocin-induced diabetic rats. However, immunohistochemical studies indicated that the water extract does not possesses insulinotropic properties. Therefore, the mechanism of this pharmacological effect includes extra-pancreatic action. However, we cannot exclude the possibility that other mechanisms may be responsible for lowering blood glucose levels. Further pharmacological and phytochemical studies are currently in progress to investigate the mode of action of this plant.

## References

[1] The Action to Control Cardiovascular Risk in Diabetes Study Group. (2008). Effects of intensive glucose lowering in type 2 diabetes. N. Engl. J. Med..

[2] Rang H.P., Dale M.M. (1999). The Endocrine System Pharmacology.

[3] Perry L.M. (1980). Medicinal Plants of East and South East Asia.

[4] Akhtar M.S., Iqbal J. (1991). Evaluation of the hypoglycaemic effect of *Achyrantes aspera* in normal and alloxan-diabetic rabbits. J. Ethnopharmacol..

[5] Frati-Munari A.C., Gordilla B.E., Altamirano P., Ariza C.R. (1988). Hypoglycaemic effect of *Opuntia streptacantha* Lemaire in NIDDM. Diabetes Care.

[6] Merat A., Sahmani M. (2003). Effect of acarbose on *in vitro* intestinal absorption of monosaccharides in diabetic rats. Arch. Iranian Med..

[7] Hirsh A.J., Yao S.Y., Young J.D. (1997). Inhibition of glucose absorption in the rat jejunum: a novel action of alpha-D-glucosidase inhibitor. Gastroenterology.

[8] Luo H., Wang L.F., Imoto T., Hiji Y. (2001). Inhibitory effect and mechanism of acarbose combined with gymnemic acid on maltose absorption in rat intestine. World J. Gastroenterol..

[9] DeFronzo R.A., Jacot E., Jequier E. (1981). The effect of insulin on the disposal of intravenous glucose. Results from indirect calorimeter and hepatic and femoral venous catheterisation. Diabetes.

[10] Risbud M.V., Bhonde R.R. (2002). Models of pancreatic regeneration in diabetes. Diabetes Res. Clin. Pract..

[11] Akowuah A.G., Amirin S., Mariam A., Aminah I. (2001). Blood sugar lowering activity of *Gynura procumbens* leaf extracts. J. Trop. Med. Plants.

[12] Chattopadhay R.R. (1999). Possible mechanism of antihyperglycaemic effect of *Azadiracta indica* leaf extract. J. Ethnopharmacol..

[13] Rosidah, Yam M.F., Sadikun A., Ahmad M., Akowuah G.A., Asmawi M.Z. (2009). Toxicology evaluation of standardized methanol extract of *Gynura procumbens*. J. Ethnopharmacol..

[14] Ahmad M., Razak A., Akowuah G.A., Asmawi Z., Zhari I. (2007). HPLC profile and antihyperglycemic effect of ethanol extracts of *Andrographis paniculata* in normal and streptozotocin-induceddiabetic rats. J. Nat. Med..

[15] Bank H.L. (1988). A quantitative enzyme-linked immunosorbent assay for rat insulin. J. Immunoassay.

[16] MacDonald M.J., Gapinski J.P. (1989). A rapid ELISA for measuring insulin in a large number of research samples. Metabolism.

[17] Wilson T.H., Wiseman G. (1954). The use of sacs of everted small intestine for the study of the transference of substances from the mucosal to the serosal surface. J. Physiol..

[18] Gray A.M., Flatt P.R. (1998). Antihyperglycemic Actions of *Eucalyptus globulus* (eucalyptus) are associated with pancreatic and extra-pancreatic effects in mice. J. Nutr..

[19] Perez C., Dominguez E., Canal J.R., Campillo J.E., Torres M.D. (2000). Hypoglycaemic activity of an aqueous extract from *Ficus Carica* (Fig Tree) leaves in streptozotocin diabetic rats. Pharm. Biol..

[20] Gray A.M., Flatt P.R. (1999). Insulin-secreting activity of the traditional antidiabetic plant *Viscum album* (mistletoe). J. Endocrinol..

[21] Plumb J.A., Milroy R., Kaye S.B. (1989). Effects of the pH dependence of 3-(4,5-dimethylthiazole-2-yl)-2,5-diphenyl-tetrazoliumbromide-formazan) absorption on chemosensitivity determined by a novel tetrazolium-based assay. Cancer Res..

